# Datasets on spatial and temporal distribution of heavy metals concentration in recent sediment at merang river system, Terengganu, Malaysia

**DOI:** 10.1016/j.dib.2020.105900

**Published:** 2020-06-21

**Authors:** Nur Marni Zaini, Hui-Wen Lee, Khairul Nizam Mohamed, Asnor Azrin Sabuti, Suhaimi Suratman, Meng-Chuan Ong

**Affiliations:** a Faculty of Science and Marine Environment, Universiti Malaysia Terengganu, 21030 Kuala Nerus, Terengganu, Malaysia; b Department of Environment, Faculty of Forestry and Environment, Universiti Putra Malaysia, 43400 Serdang, Selangor, Malaysia; c Kulliyyah of Science, International Islamic University Malaysia, 25200 Kuantan, Pahang, Malaysia; d Institute of Oceanography and Environment, Universiti Malaysia Terengganu, 21030 Kuala Nerus, Terengganu, Malaysia; e Ocean Pollution and Ecotoxicology (OPEC) Research Group, Universiti Malaysia Terengganu, 21030 Kuala Nerus, Terengganu, Malaysia

**Keywords:** Heavy metal, Merang river, South China Sea, Monsoon, Dry and wet season, sediment

## Abstract

Heavy metal pollution in an aquatic environment has become of the main concern to the world due to their non-biodegradable properties, toxicity, persistence, and their ability to adsorb into food chains. With rapid industrialization and development nowadays, heavy metals are introduced continuously into the estuaries and coastal region through rivers, runoff, and land-based point sources. These heavy metals may degrade the aquatic environment and harm the living organisms and toward human indirection through secondary contact. The dataset provided is to give an overview of the spatial and temporal distribution of the heavy metals concentration in Merang River surficial sediment collected from September 2017 to July 2018, subsequently every two months dataset. Sediment samples were collected in 44 stations along the river and 20 stations in the coastal area, which total up to 64 stations. Teflon Bomb closed digestion method with mixed acid was used to digest the sediments. The concentration of heavy metals in the sediment were analysed by using Inductively Coupled Plasma Mass Spectrometer (ICP-MS). The spatial distribution of heavy metals shows the effect of monsoon and wet and dry seasons in the sampling area. Thus, this dataset reveals six months of information on natural and anthropogenic sources intrusion at the Merang River and may also help in monitoring the pollution in the area.

**Specifications Table**

**Table:** 

**Subject**	Oceanography, analytical chemistry
**Specific subject area**	Heavy metal concentration in recent sediment at Merang River
**Type of data**	Table
**How data were acquired**	Data were acquired by detecting samples using an Inductively coupled plasma mass spectrometer (ICP-MS) ELAN 9000 after the digestion process with mixed acid.
**Data format**	Raw Data
**Parameters for data collection**	The concentration of 7 heavy metals, namely Chromium (Cr), Copper (Cu), Manganese (Mn), Lead (Pb), Iron (Fe), Arsenic (As), and Zinc (Zn) was collected subsequently every two months from September 2017 to July 2018.
**Description of data collection**	Data were collected at Merang River and coastal region; 64 sediment samples were collected, 44 samples from Merang River and 20 samples from the coastal region. Ponar Grab was used in the collection of the sediment.
**Data source location**	Merang River, Terengganu, Malaysia Latitude and longitude for each station are presented in the article
**Data accessibility**	Raw data are presented in the article

**Value of the Data**

These datasets can provide useful information about the current concentration of heavy metals to identify the concentration changes throughout time.
These datasets presented are useful information in determining the sources of heavy metals either from the natural or anthropogenic process from the Merang River towards the coastal area of Merang.
These datasets can be used by other researchers as baseline data for further research or investigation of heavy metal distribution in the Merang River and coastal area, Terengganu, Malaysia.
These datasets can also be used to monitor the interruption of anthropogenic metals from any developments in land base activities toward the river and coastal region.
These data could fill the gaps in understanding the environment, such as chemical pollutants in sediment.

## Data description

**Fig. 1:**
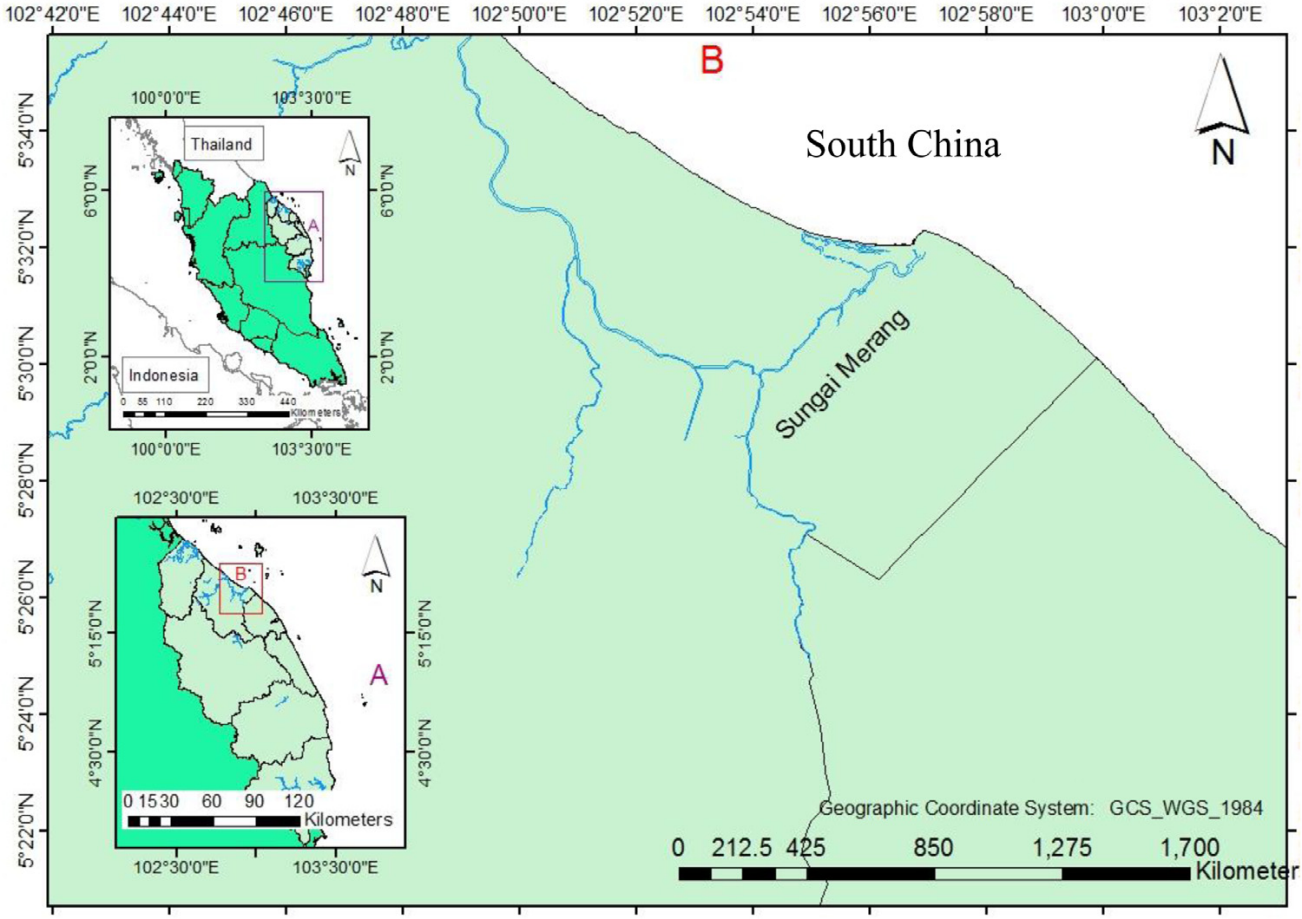
Map showing the Merang River at East Coast of Peninsular Malaysia.

Merang River situated in the district of Setiu, Terengganu, on the eastern coast of Peninsular Malaysia (Fig. 1). Merang River also faces the South China Sea in the north region of Terengganu. Merang River is one of the hotspots for tourist transportation to nearby islands such as Redang, Bidong, Lang Tengah and Perhentian Islands. Along the river, there are various types of human activities such as residential, chalets, sand mining aquaculture and agriculture. Other nearby river systems, Setiu, Marang, Kemaman and Kelantan Rivers had been intensively studied, however, best of our knowledge, there are not study especially on the pollution status of metal concentration in the sediment of the Merang River.

**Fig. 2:**
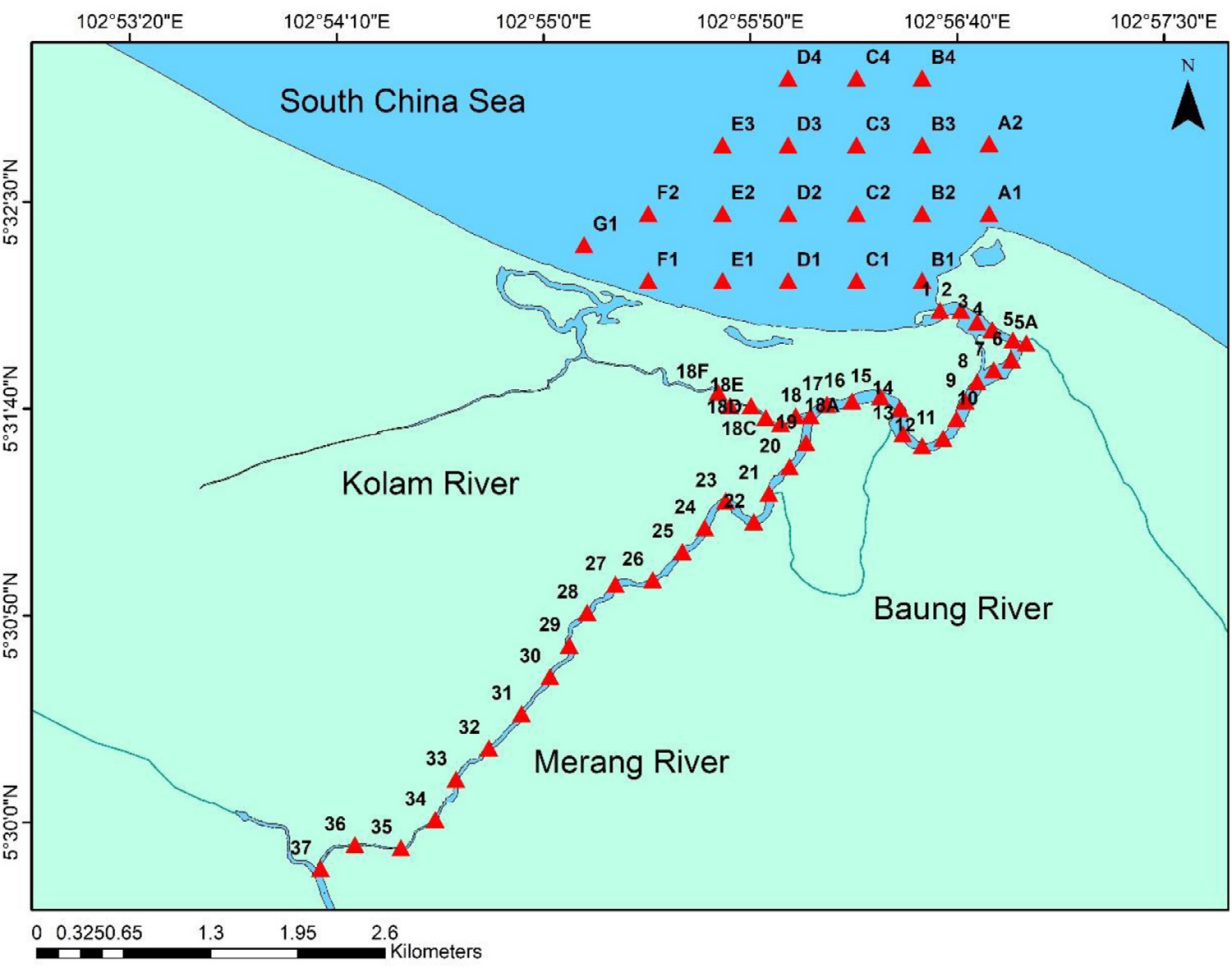
Sampling stations in Merang River and coastal area.

Surficial sediment samples were collected by using Ponar Grab from 64 stations where 44 stations at the Merang River system and 20 stations in the coastal region (Fig. 2). Coordinates of each sampling locations are shown in Table 1 below.

**Table 1:** Coordinates of sampling location at Merang River, Terengganu, Malaysia.

Station	Latitude	Longitude	Station	Latitude	Longitude
**A1**	5° 32.454′ N	102° 56.796′ E	**12**	5° 31.518′ N	102° 56.526′ E
**A2**	5° 32.736′ N	102° 56.796′ E	**13**	5° 31.566′ N	102° 56.448′ E
**B1**	5° 32.184′ N	102° 56.526′ E	**14**	5° 31.668′ N	102° 56.436′ E
**B2**	5° 32.454′ N	102° 56.526′ E	**15**	5° 31.716′ N	102° 56.358′ E
**B3**	5° 32.730′ N	102° 56.526′ E	**16**	5° 31.698′ N	102° 56.244′ E
**B4**	5° 33.000′ N	102° 56.526′ E	**17**	5° 31.686 N	102° 56.142′ E
**C1**	5° 32.184′ N	102° 56.262′ E	**18**	5° 31.638′ N	102° 56.076′ E
**C2**	5° 32.454′ N	102° 56.262′ E	**18A**	5° 31.638′ N	102° 56.016′ E
**C3**	5° 32.730′ N	102° 56.262′ E	**18B**	5° 31.608′ N	102° 55.956′ E
**C4**	5° 33.000′ N	102° 56.262′ E	**18C**	5° 31.632′ N	102° 55.896′ E
**D1**	5° 32.184′ N	102° 55.986′ E	**18D**	5° 31.680′ N	102° 55.836′ E
**D2**	5° 32.454′ N	102° 55.986′ E	**18E**	5° 31.680′ N	102° 55.752′ E
**D3**	5° 32.730′ N	102° 55.986′ E	**18F**	5° 31.734′ N	102° 55.704′ E
**D4**	5° 33.000′ N	102° 55.986′ E	**19**	5° 31.530′ N	102° 56.058′ E
**E1**	5° 32.184′ N	102° 55.722′ E	**20**	5° 31.434′ N	102° 55.992′ E
**E2**	5° 32.454′ N	102° 55.722′ E	**21**	5° 31.326′ N	102° 55.908′ E
**E3**	5° 32.730′ N	102° 55.722′ E	**22**	5° 31.212′ N	102° 55.848′ E
**F1**	5° 32.184′ N	102° 55.422′ E	**23**	5° 31.296′ N	102° 55.734′ E
**F2**	5° 32.454′ N	102° 55.422′ E	**24**	5° 31.188′ N	102° 55.650′ E
**G1**	5° 32.328′ N	102° 55.164′ E	**25**	5° 31.092′ N	102° 55.560′ E
**1**	5° 32.064′ N	102° 56.598′ E	**26**	5° 30.978′ N	102° 55.440′ E
**2**	5° 32.064′ N	102° 56.682′ E	**27**	5° 30.960′ N	102° 55.290′ E
**3**	5° 32.016′ N	102° 56.748′ E	**28**	5° 30.846′ N	102° 55.176′ E
**4**	5° 31.986′ N	102° 56.808′ E	**29**	5° 30.714′ N	102° 55.104′ E
**5**	5° 31.944′ N	102° 56.892′ E	**30**	5° 30.588′ N	102° 55.026′ E
**5A**	5° 31.932′ N	102° 56.946′ E	**31**	5° 30.438′ N	102° 54.912′ E
**6**	5° 31.866′ N	102° 56.886′ E	**32**	5° 30.300′ N	102° 54.780′ E
**7**	5° 31.824′ N	102° 56.814′ E	**33**	5° 30.174′ N	102° 54.648′ E
**8**	5° 31.776′ N	102° 56.748′ E	**34**	5° 30.012′ N	102° 54.564′ E
**9**	5° 31.698′ N	102° 56.700′ E	**35**	5° 29.898′ N	102° 54.426′ E
**10**	5° 31.626′ N	102° 56.664′ E	**36**	5° 29.910′ N	102° 54.240′ E
**11**	5° 31.548′ N	102° 56.610′ E	**37**	5° 29.814′ N	102° 54.102′ E

The certified standard from Standard Reference Material 1646a Estuarine Sediment was used to check the accuracy and precision of the sample analysis procedures [1]. The concentration values measured in the standard shown in Table 2 exhibit a good recovery percentage within ±3% of the certified values.

**Table 2:** Measured value and percentage recovery test of heavy metals analysis.

Element	Measured value (mg/kg dry weights)	Certified value (mg/kg dry weights)	Recovery Test (%)
**Li**	16.8 ± 1.3	18	93.3
**Cr**	39.26 ± 1.2	40.9 ± 1.9	96.0
**Mn**	226.29 ± 0.9	234.5 ± 2.8	96.5
Fe*	1.91 ± 0.58	2.008 ± 0.039	95.1
**Cu**	9.87 ± 0.28	10.01 ± 0.34	98.6
**Zn**	49.39 ± 1.04	48.9 ± 1.6	101
**As**	6.06 ± 0.56	6.23 ± 0.21	97.3
**Pb**	11.58 ± 0.45	11.7 ± 1.2	99.0

⁎ concentration of Fe is in%.

Sampling was conducted in September 2017 to July 2018 for subsequently every two months to represent all the seasonally season. The concentration of heavy metals that has been determined during the sampling period was recorded in Table 3 to Table 8. Generally, recorded data shows the influence of monsoon and dry and wet seasons affecting the distribution of heavy metals concentration in the sediment at the Merang River and coastal region. High concentration was observed in stations in coastal areas due to runoff from river flushing off sediment from upstream to downstream and towards coastal regions [2,3].

**Table 3:** Heavy metals concentration dataset: September 2017 (Dry season).

Station	Concentration (mg/kg dry weights)							
	Li	Cr	Mn	Fe*	Cu	Zn	As	Pb
**A1**	15.3	15.3	387	3.21	14.1	57.1	3.76	33.5
**A2**	14.3	16.7	430	2.28	24.3	54.2	3.91	25.6
**B1**	13.2	17.6	345	1.35	21.4	36.9	1.99	17.6
**B2**	14.8	30.8	436	3.95	18.6	69.7	1.97	34.3
**B3**	10.1	23.8	527	3.91	18.2	62.1	1.78	25.3
**B4**	15.7	16.8	567	2.22	17.3	48.5	1.82	26.1
**C1**	17.1	18.4	833	3.21	12.9	50.8	3.21	28.7
**C2**	18.5	20.0	777	1.21	12.8	87.2	2.47	42.0
**C3**	25.0	33.7	679	1.74	18.4	86.3	3.08	35.9
**C4**	16.9	16.2	581	2.26	18.3	57.9	1.96	29.9
**D1**	21.0	9.7	603	2.20	8.6	28.7	1.39	17.6
**D2**	20.5	7.5	562	1.72	11.7	45.8	1.3	14.0
**D3**	20.0	27.3	753	1.20	19.3	62.8	2.18	34.9
**D4**	24.8	25.6	469	1.44	24.9	77.2	3.56	24.4
**E1**	19.7	12.5	523	1.67	17.0	60.9	1.27	13.9
**E2**	14.6	13.3	832	3.13	20.0	44.7	2.36	23.6
**E3**	10.4	18.6	557	3.45	20.4	36.1	1.41	24.6
**F1**	11.9	7.1	416	1.48	12.7	40.4	0.98	11.8
**F2**	10.4	21.3	616	2.28	11.2	46.2	1.00	15.7
**G1**	7.04	35.4	506	1.89	13.1	52.0	1.17	15.0
**1**	7.00	14.5	358	2.64	16.3	45.9	2.30	13.4
**2**	5.65	21.5	185	1.09	12.6	39.7	1.04	11.7
**3**	10.8	28.4	462	1.87	13.0	42.8	3.00	12.5
**4**	16.2	23.0	632	1.14	13.4	41.3	3.82	8.92
**5**	22.7	13.8	524	0.42	12.3	42.0	3.41	5.31
**5A**	29.2	31.6	416	0.62	9.9	44.1	2.35	6.72
**6**	9.36	12.5	81.8	0.82	11.9	46.2	1.29	8.12
**7**	8.00	10.4	71.8	0.73	12.5	28.0	1.12	7.34
**8**	3.68	5.0	66.9	0.40	5.2	7.5	0.81	3.19
**9**	4.39	7.2	38.1	0.50	9.7	6.7	0.90	3.65
**10**	5.66	12.1	64.1	0.66	11.3	15.1	0.60	4.35
**11**	4.48	6.3	38.0	0.51	10.0	11.4	0.68	6.44
**12**	4.26	6.4	34.0	0.36	8.7	26.2	0.83	2.56
**13**	15.7	12.1	154	0.81	11.1	41.1	1.71	8.84
**14**	20.2	9.3	159	0.83	13.4	53.2	1.92	17.9
**15**	11.4	8.0	165	0.85	8.7	43.5	2.74	7.29
**16**	13.6	6.7	45.7	0.64	5.6	20.8	2.12	5.37
**17**	15.7	8.0	152	0.88	7.7	30.9	2.77	7.87
**18**	8.22	8.4	112	1.25	9.8	21.7	3.15	10.6
**18A**	5.02	8.2	89.2	0.79	11.4	24.2	1.84	7.25
**18B**	8.44	8.0	208	0.64	7.3	26.8	2.31	8.84
**18C**	6.88	9.1	66.4	0.46	9.5	14.7	2.35	9.32
**18D**	10.0	11.8	86.5	0.33	11.7	20.0	1.91	4.76
**18E**	9.31	19.5	131	0.72	11.3	25.3	2.61	12.8
**18F**	8.66	24.5	141	0.84	3.2	25.9	2.80	13.6
**19**	7.34	14.5	70.1	0.73	8.9	22.7	2.47	8.98
**20**	3.07	7.6	42.4	0.40	8.5	19.7	1.67	3.52
**21**	5.53	32.6	50.8	0.64	12.8	16.8	2.32	7.58
**22**	4.69	23.2	33.4	0.47	11.7	14.5	2.44	5.04
**23**	4.47	9.5	43.1	0.53	7.5	13.4	1.87	4.83
**24**	5.24	12.1	96.0	0.51	8.4	19.2	2.61	4.43
**25**	6.00	4.9	64.2	0.50	16.5	10.0	1.43	2.34
**26**	5.25	13.2	43.3	0.69	5.6	10.8	2.72	7.74
**27**	2.56	24.9	21.3	0.62	3.6	17.7	1.27	3.26
**28**	9.46	36.5	66.4	0.92	1.7	24.6	2.83	7.14
**29**	5.12	30.5	41.9	0.71	8.1	16.6	2.13	6.19
**30**	5.32	24.4	118	0.72	2.8	8.5	2.32	6.23
**31**	5.52	20.4	43.4	0.61	6.5	10.6	2.28	7.01
**32**	4.32	15.8	29.4	0.48	3.1	10.7	1.78	5.26
**33**	8.56	11.7	89.4	0.57	5.7	5.1	2.45	5.45
**34**	4.70	12.4	52.3	0.75	8.8	4.1	1.78	6.31
**35**	4.38	5.0	41.5	0.90	7.2	6.0	1.77	5.19
**36**	3.82	23.3	46.5	0.67	8.0	5.8	1.49	5.17
**37**	6.22	20.6	52.4	0.51	8.7	4.0	1.73	5.94

⁎ All concentration is in mg/kg dry weights, except for Fe which is in%.

**Table 4:** Heavy metals concentration dataset: November 2017 (Wet season).

Station	Concentration (mg/kg dry weights)							
	Li	Cr	Mn	Fe	Cu	Zn	As	Pb
**A1**	37.5	5.96	367	2.10	17.7	53.2	2.52	47.5
**A2**	48.4	7.02	306	2.50	19.9	64.2	2.81	52.7
**B1**	45.7	5.08	278	1.79	16.0	42.2	2.22	42.3
**B2**	42.9	3.14	250	1.08	10.9	27.3	1.63	31.8
**B3**	35.1	11.9	297	2.12	15.8	57.8	2.60	45.8
**B4**	22.0	4.78	222	1.61	10.8	37.2	1.42	27.9
**C1**	17.1	8.34	215	0.79	6.60	26.0	1.82	26.6
**C2**	18.4	6.85	197	0.93	7.09	22.3	1.43	28.0
**C3**	29.2	5.35	267	1.62	12.0	29.7	1.99	36.9
**C4**	34.0	5.31	313	2.16	13.9	41.7	2.10	39.3
**D1**	15.5	5.50	407	0.78	11.8	24.3	1.16	32.5
**D2**	17.1	6.63	245	0.81	6.85	33.7	0.99	18.9
**D3**	22.8	17.2	233	1.40	12.3	38.8	1.61	28.5
**D4**	26.2	5.84	277	1.75	11.8	28.0	1.55	32.5
**E1**	12.1	11.7	263	1.26	9.83	38.0	2.41	9.52
**E2**	16.7	7.84	250	0.77	7.86	27.7	0.82	19.7
**E3**	16.3	3.31	246	0.81	5.57	17.0	0.96	17.3
**F1**	18.1	6.69	196	0.69	6.88	5.77	1.12	21.0
**F2**	19.5	9.90	232	0.79	6.32	13.0	0.77	17.9
**G1**	18.5	5.28	207	0.75	5.71	7.11	1.55	20.2
**1**	12.5	11.0	220	0.56	6.08	35.8	1.69	8.82
**2**	14.4	5.07	220	0.37	6.81	28.8	1.58	17.3
**3**	34.0	8.16	220	1.69	6.45	32.3	1.64	13.1
**4**	65.7	7.97	153	1.20	6.98	31.9	1.50	13.3
**5**	56.0	7.78	85.8	0.71	7.51	31.4	1.35	13.6
**5A**	46.4	7.32	107	0.53	5.73	32.6	0.98	9.80
**6**	13.6	9.51	16.1	0.35	3.94	33.8	0.61	6.00
**7**	14.0	11.7	16.6	0.10	5.22	57.7	0.74	2.47
**8**	14.4	2.88	34.1	0.20	6.49	20.7	0.87	9.56
**9**	12.5	3.01	13.5	0.19	2.18	45.1	2.04	4.24
**10**	12.1	7.88	12.0	0.10	1.95	46.8	1.12	4.19
**11**	12.6	7.66	28.0	0.10	2.66	41.2	0.95	3.92
**12**	13.0	7.44	30.0	0.05	1.14	44.6	1.21	2.87
**13**	12.0	6.58	49.1	0.08	4.65	18.9	0.72	11.8
**14**	11.1	5.71	17.7	0.11	3.78	44.3	1.31	5.08
**15**	13.4	7.84	17.6	0.15	2.51	28.0	2.38	7.25
**16**	12.0	3.76	15.8	0.19	4.57	29.8	1.84	6.85
**17**	15.0	9.08	12.6	0.14	2.34	37.0	2.04	9.92
**18**	16.9	12.3	20.1	0.51	3.35	31.3	2.62	8.43
**18A**	21.2	4.64	16.1	0.31	5.63	36.8	3.19	14.6
**18B**	19.7	6.11	12.1	0.05	2.49	42.3	3.03	4.05
**18C**	13.2	5.27	14.0	0.11	2.95	43.1	2.58	7.98
**18D**	15.5	2.68	15.8	0.07	2.81	36.5	2.72	5.94
**18E**	16.1	8.00	24.3	0.08	3.04	32.7	4.11	6.55
**18F**	16.7	8.13	21.8	0.11	3.23	19.5	3.64	14.8
**19**	12.8	5.66	19.2	0.14	3.40	36.0	1.41	4.68
**20**	13.3	3.18	18.2	0.13	2.02	40.3	1.94	5.71
**21**	15.0	4.74	20.6	0.12	4.72	20.7	0.97	4.34
**22**	15.0	7.38	23.8	0.16	2.14	39.6	0.85	6.06
**23**	10.5	5.22	13.9	0.12	2.04	44.4	0.72	3.60
**24**	11.8	3.05	11.4	0.15	1.93	49.1	0.76	4.41
**25**	11.3	5.04	16.6	0.19	4.76	29.2	0.66	5.75
**26**	10.8	12.1	9.78	0.11	1.23	56.1	1.37	3.84
**27**	10.5	2.48	10.2	0.09	2.55	57.7	1.49	3.38
**28**	12.1	5.40	13.6	0.23	2.35	41.7	1.60	4.92
**29**	12.8	11.1	12.2	0.14	2.61	38.8	1.20	5.30
**30**	12.7	15.9	6.55	0.09	0.61	65.3	0.79	2.05
**31**	12.6	11.0	11.8	0.11	1.37	54.4	0.90	3.94
**32**	11.7	5.98	8.90	0.09	1.99	37.0	1.67	2.93
**33**	13.0	9.68	20.0	0.16	4.88	18.0	1.63	5.38
**34**	14.4	7.83	15.6	0.12	3.58	44.7	1.18	3.70
**35**	13.7	10.3	10.3	0.17	2.27	71.3	0.72	2.02
**36**	12.9	12.7	11.7	0.14	1.70	43.7	0.55	5.01
**37**	11.3	17.2	5.52	0.03	3.17	82.5	1.37	1.00

*All concentration is in mg/kg dry weights, except for Fe which is in%.

**Table 5:** Heavy metals concentration dataset: January 2018 (Wet season).

Station	Concentration (mg/kg dry weights)							
	Li	Cr	Mn	Fe*	Cu	Zn	As	Pb
**A1**	16.5	70.7	658	3.13	36.9	71.3	4.37	46.8
**A2**	21.2	57.7	612	3.04	37.7	70.4	4.06	42.9
**B1**	17.7	45.8	703	3.21	26.6	72.1	3.75	29.9
**B2**	14.2	33.9	521	2.87	28.2	68.7	4.49	39.6
**B3**	18.6	45.9	339	2.54	40.6	65.4	5.65	41.3
**B4**	17.2	69.6	257	2.82	38.1	46.4	4.49	42.9
**C1**	14.0	85.5	422	2.25	18.9	37.9	3.03	27.3
**C2**	10.7	33.4	443	3.39	18.2	55.0	3.25	25.8
**C3**	19.7	42.6	464	3.30	19.7	59.0	3.07	26.9
**C4**	14.8	51.7	464	3.21	23.0	62.9	2.89	28.1
**D1**	13.0	37.5	464	3.08	33.6	56.4	2.87	29.8
**D2**	11.2	23.2	662	3.35	19.3	49.8	3.49	27.6
**D3**	17.2	24.8	861	2.81	24.7	54.8	3.17	28.7
**D4**	14.8	26.4	780	2.27	23.9	46.0	3.05	35.5
**E1**	12.8	27.4	467	1.94	12.7	37.3	3.22	19.8
**E2**	10.7	26.9	362	2.59	15.7	32.3	3.39	25.8
**E3**	12.1	46.0	255	3.23	18.4	57.5	2.65	29.8
**F1**	11.2	65.1	249	3.35	22.4	67.2	3.27	27.7
**F2**	16.3	29.9	305	3.96	25.7	76.8	2.96	29.5
**G1**	20.0	21.8	362	3.23	26.6	59.2	2.26	38.5
**1**	7.20	6.42	340	1.17	7.47	41.6	1.56	17.3
**2**	6.45	10.1	383	1.42	7.07	52.9	1.29	14.0
**3**	7.25	9.66	316	1.65	11.4	16.1	1.78	21.0
**4**	8.04	8.28	280	1.54	9.68	28.6	2.50	19.1
**5**	9.86	11.3	242	1.21	14.7	17.2	3.73	23.7
**5A**	10.8	14.3	204	0.88	19.8	19.7	4.96	28.4
**6**	12.5	24.4	144	1.45	16.6	22.3	2.61	11.1
**7**	3.88	15.0	100	1.04	12.3	25.2	1.65	8.45
**8**	5.44	18.4	56.3	0.63	8.09	19.4	0.69	5.81
**9**	6.75	21.8	98.4	0.61	12.4	6.00	1.19	11.5
**10**	8.06	8.84	73.0	0.75	7.21	8.88	1.91	9.02
**11**	5.28	8.82	38.6	0.37	3.30	19.1	0.51	6.54
**12**	6.10	4.73	29.0	0.25	3.04	23.6	0.74	4.04
**13**	6.44	6.55	62.7	0.54	7.20	19.3	0.76	10.3
**14**	7.82	3.46	47.6	0.32	4.16	14.9	0.75	6.75
**15**	5.98	2.70	49.1	0.35	5.04	10.5	1.95	7.01
**16**	4.50	2.01	42.9	0.30	5.27	5.13	0.8	6.55
**17**	5.32	2.80	46.8	0.36	4.95	8.34	1.17	6.37
**18**	7.64	5.89	50.6	0.40	4.36	11.6	0.95	6.99
**18A**	2.36	2.16	20.6	0.36	5.23	21.8	0.84	7.13
**18B**	6.98	1.51	39.9	0.32	6.09	12.9	0.73	7.27
**18C**	2.94	1.45	14.9	0.24	3.31	29.6	1.06	6.00
**18D**	3.90	2.38	29.5	0.15	3.85	20.7	1.38	4.74
**18E**	4.36	3.03	49.9	0.26	4.02	31.7	1.16	6.16
**18F**	7.18	3.68	74.5	0.36	4.19	20.3	0.94	7.57
**19**	6.98	8.12	39.5	0.51	4.00	14.9	1.16	6.05
**20**	5.87	9.84	65.8	0.35	4.77	9.48	0.77	5.94
**21**	5.09	10.9	23.6	0.30	2.91	8.50	0.82	3.76
**22**	4.23	7.26	22.2	0.25	2.45	18.5	0.89	2.94
**23**	8.24	11.0	30.1	0.85	3.69	11.4	0.87	7.09
**24**	10.2	4.71	38.1	0.27	4.93	15.5	0.58	5.01
**25**	9.10	5.47	52.4	0.56	6.49	24.0	0.59	6.36
**26**	6.85	6.23	44.9	0.52	7.05	32.5	0.81	6.77
**27**	5.64	13.1	41.6	0.41	3.78	26.4	0.59	5.69
**28**	4.00	19.9	19.8	0.29	2.42	20.2	0.64	2.62
**29**	3.94	23.9	29.3	0.33	4.22	17.9	0.51	3.37
**30**	4.68	20.9	15.3	0.20	1.87	9.62	0.43	2.40
**31**	4.88	18.0	18.9	0.24	2.53	21.5	0.37	2.43
**32**	4.88	22.1	24.0	0.16	3.08	14.0	0.67	3.26
**33**	2.20	26.2	40.9	0.23	9.08	14.9	0.27	1.69
**34**	2.04	16.9	11.0	0.36	11.0	16.9	0.39	1.39
**35**	3.20	7.66	18.7	0.48	16.3	23.2	0.49	3.13
**36**	3.59	12.1	54.3	0.40	14.9	22.2	0.69	5.35
**37**	3.98	5.16	14.8	0.44	13.5	21.5	0.59	1.50

⁎ All concentration is in mg/kg dry weights, except for Fe which is in%.

**Table 6:** Heavy metals concentration dataset: March 2018 (Wet season).

Station	Concentration (mg/kg dry weights)							
	Li	Cr	Mn	Fe*	Cu	Zn	As	Pb
**A1**	13.7	24.0	655	2.75	11.8	45.3	2.42	27.2
**A2**	21.2	32.3	540	2.31	29.9	66.6	5.48	24.1
**B1**	17.5	21.6	426	1.86	16.8	87.9	1.93	21.1
**B2**	19.4	10.8	409	1.62	27.5	77.2	1.40	14.8
**B3**	21.4	13.2	870	1.74	38.2	82.5	4.09	49.7
**B4**	22.4	16.0	679	2.09	22.0	73.2	3.37	45.5
**C1**	12.4	18.7	482	2.43	18.2	70.2	2.13	25.3
**C2**	15.6	19.6	731	3.94	14.2	67.3	2.41	37.4
**C3**	15.3	27.0	460	3.19	23.7	97.6	4.30	52.2
**C4**	14.9	16.0	689	1.95	16.4	60.5	3.01	36.1
**D1**	15.1	14.8	635	1.34	11.9	79.1	1.39	15.2
**D2**	16.0	11.2	580	2.09	7.43	69.8	1.71	17.4
**D3**	16.9	19.9	709	1.21	15.4	57.6	2.89	37.5
**D4**	17.0	16.0	792	1.48	17.9	75.4	3.17	39.3
**E1**	13.6	7.84	431	1.75	6.68	55.2	1.58	16.0
**E2**	10.3	10.8	842	2.71	13.6	35.1	2.08	23.0
**E3**	12.6	13.8	629	3.61	11.4	45.2	2.51	28.1
**F1**	11.5	15.7	781	1.88	7.13	40.2	1.67	15.8
**F2**	13.4	17.6	548	2.04	8.46	46.1	2.00	18.6
**G1**	15.4	28.3	785	3.09	13.3	52.0	2.97	28.7
**1**	11.2	26.3	595	2.45	7.71	39.2	3.58	12.5
**2**	13.1	21.8	405	1.80	10.9	23.4	3.87	23.4
**3**	14.3	17.3	632	2.12	34.1	31.3	5.12	44.6
**4**	20.8	36.2	686	1.96	31.5	16.8	11.5	66.8
**5**	23.2	26.7	535	2.04	37.6	12.7	7.84	44.9
**5A**	10.7	17.2	193	2.12	8.96	8.60	6.10	15.4
**6**	15.2	26.5	186	1.59	7.93	9.54	4.36	22.2
**7**	8.58	20.4	82.2	1.06	6.89	10.5	1.36	9.46
**8**	8.16	18.4	60.3	0.41	5.37	8.44	1.41	6.49
**9**	9.32	21.4	88.3	0.75	5.44	18.1	1.36	7.73
**10**	12.3	14.4	83.2	0.56	5.50	4.91	1.71	10.0
**11**	11.8	9.72	48.0	0.38	6.37	12.0	1.24	6.34
**12**	11.3	6.12	65.6	0.60	5.66	11.8	3.24	7.72
**13**	9.60	6.05	56.8	0.78	9.21	14.1	1.68	12.4
**14**	9.12	5.98	61.2	0.96	7.44	5.62	1.30	8.27
**15**	4.96	16.05	59.0	1.14	8.33	22.0	0.89	5.05
**16**	5.14	14.6	50.1	0.42	4.12	17.6	1.07	5.87
**17**	9.96	26.7	69.1	0.68	8.08	4.44	1.52	8.25
**18**	9.34	6.87	65.5	0.84	7.15	9.10	3.31	9.46
**18A**	8.22	7.33	58.5	0.52	4.95	8.66	1.63	9.58
**18B**	10.2	20.4	57.0	0.73	6.31	21.8	1.64	14.0
**18C**	9.20	24.5	55.4	0.48	6.20	39.0	1.42	9.62
**18D**	5.48	9.68	36.5	0.24	2.32	22.1	0.43	5.78
**18E**	5.16	6.61	54.7	0.30	3.17	14.4	1.07	6.09
**18F**	6.84	23.4	48.6	0.49	4.70	12.7	0.83	8.05
**19**	5.72	8.84	32.1	0.40	2.85	13.2	1.17	4.81
**20**	6.33	15.1	37.0	0.52	3.71	17.1	1.06	6.24
**21**	8.28	14.1	60.2	0.54	5.38	5.80	1.13	6.84
**22**	7.45	13.2	48.0	0.53	4.13	10.2	0.87	6.30
**23**	10.8	17.8	53.7	0.60	4.26	14.6	1.16	6.63
**24**	11.4	6.37	47.1	0.56	4.34	10.3	1.55	7.21
**25**	12.0	8.22	65.6	0.66	5.17	9.62	1.52	10.6
**26**	8.16	9.52	64.3	0.77	4.79	9.10	1.41	8.64
**27**	9.48	7.10	49.1	0.94	4.46	7.59	1.29	8.43
**28**	7.84	14.1	46.6	0.80	5.62	14.3	2.08	8.04
**29**	6.06	22.3	71.5	0.64	4.94	16.3	0.61	6.01
**30**	7.00	13.3	39.5	0.55	4.26	4.15	0.74	5.99
**31**	6.04	7.35	47.8	0.58	3.63	21.3	0.56	6.61
**32**	6.98	8.22	33.6	0.29	3.80	11.4	0.98	5.16
**33**	5.49	8.90	34.3	0.32	3.91	19.4	0.61	4.53
**34**	6.64	10.5	69.5	0.39	3.09	12.1	0.86	5.61
**35**	7.92	16.6	49.9	1.12	5.75	15.4	1.09	8.24
**36**	8.30	14.3	59.7	1.38	7.17	3.49	1.08	9.34
**37**	13.9	15.4	54.8	0.77	8.58	3.59	0.70	11.4

*All concentration is in mg/kg dry weights, except for Fe which is in%.

**Table 7:** Heavy metals concentration dataset: May 2018 (Dry season).

Station	Concentration (mg/kg dry weights)							
	Li	Cr	Mn	Fe*	Cu	Zn	As	Pb
**A1**	14.0	40.8	597	2.50	9.57	37.5	1.96	24.4
**A2**	19.7	45.2	546	3.14	13.5	40.6	2.18	33.0
**B1**	13.7	26.1	378	1.53	6.66	75.3	1.75	21.7
**B2**	10.5	14.9	611	2.36	8.38	68.3	1.67	23.0
**B3**	13.4	28.2	774	2.83	19.5	74.0	3.29	48.7
**B4**	16.2	33.1	525	2.39	13.2	43.3	1.99	30.7
**C1**	12.8	14.2	592	1.94	8.91	58.6	1.88	23.9
**C2**	11.2	44.5	544	2.05	9.01	62.6	1.82	25.3
**C3**	19.8	60.4	779	2.00	19.4	66.5	2.71	45.4
**C4**	15.5	17.0	641	2.18	14.7	47.3	2.67	36.3
**D1**	10.2	29.7	749	2.36	9.19	78.6	1.55	20.2
**D2**	11.0	32.1	695	1.99	15.5	61.5	2.56	38.2
**D3**	10.6	34.4	722	1.62	21.8	89.8	3.57	56.2
**D4**	19.1	30.7	882	1.94	22.5	74.2	3.05	49.7
**E1**	27.5	21.1	463	2.26	7.58	82.0	1.93	24.2
**E2**	21.0	15.1	462	2.38	9.26	61.0	1.45	22.2
**E3**	12.5	20.2	461	3.11	14.1	39.9	2.08	33.9
**F1**	13.8	32.2	584	2.56	11.8	50.4	1.06	17.9
**F2**	15.1	28.1	706	2.04	9.40	42.8	1.70	22.0
**G1**	14.4	36.7	765	3.05	12.8	35.1	2.41	32.5
**1**	15.4	16.7	710	3.14	6.66	47.2	2.06	16.7
**2**	16.5	16.9	655	2.22	10.3	59.3	2.23	37.1
**3**	13.9	24.4	554	1.75	13.9	64.4	2.15	35.0
**4**	14.0	52.9	504	1.27	18.1	39.8	1.88	41.2
**5**	11.6	27.1	416	1.79	9.02	15.1	1.61	15.7
**5A**	11.5	42.4	328	1.70	16.12	10.3	1.65	32.0
**6**	11.3	70.2	132	1.61	10.8	5.54	1.68	13.9
**7**	10.1	85.2	92.0	0.88	8.22	6.18	0.88	11.7
**8**	11.4	63.1	63.5	0.57	8.09	9.74	1.06	8.72
**9**	12.7	62.8	76.7	0.87	10.4	7.69	0.93	16.0
**10**	7.50	55.3	60.5	0.51	6.10	19.6	0.90	11.9
**11**	9.34	37.0	25.8	0.28	3.17	23.8	0.54	3.42
**12**	11.2	79.2	102	0.51	9.04	37.2	1.10	9.68
**13**	9.38	88.8	73.4	0.75	10.0	4.51	0.79	8.22
**14**	8.80	98.0	103	0.64	6.85	14.6	0.90	6.65
**15**	7.58	74.9	75.1	0.56	9.77	17.4	0.89	8.32
**16**	9.32	24.6	40.9	0.50	5.07	12.3	0.56	10.2
**17**	14.0	30.3	59.4	0.82	8.08	13.2	1.36	14.4
**18**	7.58	28.2	72.1	0.62	5.01	14.1	0.81	6.95
**18A**	7.06	31.7	46.3	0.41	4.86	5.07	0.33	6.25
**18B**	11.4	26.0	61.5	0.61	5.89	10.3	0.87	13.0
**18C**	10.6	20.2	95.4	0.51	3.48	20.8	0.62	8.00
**18D**	10.9	12.9	48.4	0.45	4.87	5.58	0.60	9.72
**18E**	13.8	16.6	54.5	0.48	5.36	8.42	0.83	18.5
**18F**	12.2	14.7	60.4	0.50	4.39	22.3	1.26	8.39
**19**	10.6	15.6	80.0	0.80	6.17	12.3	0.82	8.38
**20**	12.1	15.6	35.0	0.52	2.87	19.9	1.29	6.72
**21**	13.5	15.6	43.4	0.58	6.33	17.70	0.83	13.5
**22**	14.5	18.3	40.2	0.74	6.83	14.3	0.69	17.5
**23**	14.7	21.0	59.3	0.65	4.64	11.0	1.04	7.72
**24**	14.9	30.5	42.3	0.62	8.74	16.9	1.87	13.0
**25**	15.2	21.8	43.2	0.69	5.45	10.4	0.86	10.3
**26**	15.4	24.6	41.9	0.72	6.92	7.05	1.13	13.7
**27**	11.0	30.3	38.6	0.60	5.29	17.1	0.86	11.0
**28**	10.5	35.9	44.4	0.84	6.99	8.6	1.05	11.0
**29**	9.72	10.2	29.1	0.60	3.82	14.3	0.61	8.76
**30**	9.76	24.3	29.3	0.58	6.10	6.75	0.50	12.5
**31**	9.80	41.9	40.6	0.53	8.61	13.6	0.51	11.2
**32**	9.24	59.4	54.2	0.51	7.50	19.0	0.51	6.38
**33**	12.6	14.9	41.7	0.32	6.51	7.9	0.83	12.6
**34**	12.1	10.8	30.9	0.44	5.03	18.7	0.50	11.8
**35**	12.1	12.8	37.9	0.46	7.54	14.5	0.70	14.1
**36**	5.64	17.6	43.3	0.47	4.78	28.5	0.43	4.35
**37**	7.46	22.4	34.7	0.46	3.12	24.3	0.77	6.33

*All concentration is in mg/kg dry weights, except for Fe which is in%.

**Table 8:** Heavy metals concentration dataset: July 2018 (Dry season).

Station	Concentration (mg/kg dry weights)							
	Li	Cr	Mn	Fe*	Cu	Zn	As	Pb
**A1**	12.6	6.83	479	2.36	10.9	14.4	1.79	25.6
**A2**	17.4	5.45	490	3.78	18.7	30.8	1.67	29.4
**B1**	15.5	4.06	501	3.28	16.5	25.9	1.72	28.7
**B2**	13.7	7.38	513	2.77	14.3	24.4	1.76	27.9
**B3**	13.8	10.7	397	3.29	14.0	22.9	1.65	26.9
**B4**	12.5	11.9	413	3.43	13.3	14.4	1.19	22.2
**C1**	10.5	11.1	295	1.65	9.94	14.5	1.38	21.1
**C2**	13.7	10.8	365	1.98	9.86	14.7	1.28	20.3
**C3**	12.6	13.2	390	2.85	14.2	17.5	1.50	22.7
**C4**	16.3	11.2	369	3.11	14.1	14.2	1.44	23.9
**D1**	14.7	16.7	246	1.18	6.65	12.4	1.12	17.6
**D2**	13.2	22.2	456	1.62	9.00	16.1	1.09	13.3
**D3**	12.3	13.0	384	1.89	14.8	19.8	1.59	25.7
**D4**	16.2	24.7	541	2.16	19.1	31.7	1.77	30.3
**E1**	12.0	8.62	756	2.08	13.1	45.7	1.30	23.2
**E2**	20.6	18.6	627	2.71	15.4	20.6	1.39	28.3
**E3**	13.1	15.8	537	3.05	15.7	35.1	1.17	27.0
**F1**	12.9	16.4	606	1.49	9.74	24.8	0.91	19.4
**F2**	12.7	17.0	428	1.89	8.58	14.5	1.09	18.8
**G1**	12.7	19.1	500	1.91	10.1	18.9	1.63	19.4
**1**	12.8	10.2	256	2.22	12.6	21.6	1.89	28.0
**2**	11.0	15.5	240	1.04	9.66	16.7	1.29	21.8
**3**	16.6	20.7	243	1.38	10.0	13.9	1.33	23.0
**4**	14.1	9.30	246	1.71	10.3	11.1	1.36	24.2
**5**	12.3	8.80	151	1.24	9.42	11.5	0.92	19.9
**5A**	11.5	29.8	50.8	0.84	7.60	18.8	0.61	17.8
**6**	11.9	14.2	135	0.64	9.16	6.36	1.56	14.7
**7**	6.38	13.5	51.6	0.43	9.58	25.0	1.29	16.3
**8**	7.46	6.43	68.5	0.44	3.97	26.7	1.01	16.2
**9**	12.0	11.1	50.2	0.47	7.47	6.77	0.57	16.0
**10**	13.5	27.5	58.5	0.64	8.68	8.60	0.68	19.6
**11**	12.4	19.8	9.26	0.52	7.68	10.8	0.65	18.0
**12**	11.3	12.1	45.8	0.39	6.68	12.9	0.62	16.5
**13**	13.2	17.3	20.0	0.30	8.50	28.1	0.50	17.1
**14**	15.0	22.5	54.0	0.39	10.3	19.7	0.51	17.6
**15**	5.90	27.3	52.5	0.57	6.67	22.4	0.47	12.9
**16**	5.28	24.9	78.8	0.68	8.50	26.3	0.42	8.12
**17**	4.26	22.0	34.3	0.65	8.02	29.9	0.42	10.4
**18**	9.82	19.1	48.0	0.61	7.55	7.71	0.63	12.8
**18A**	15.6	10.9	60.7	0.51	8.80	7.35	0.62	23.3
**18B**	7.44	26.8	30.3	0.22	5.63	23.2	0.18	18.0
**18C**	3.52	22.8	7.20	0.07	7.33	22.8	0.08	19.3
**18D**	13.2	18.8	29.9	0.36	9.02	6.97	0.32	20.5
**18E**	11.8	15.8	26.1	0.29	8.72	8.20	0.26	17.6
**18F**	5.18	12.7	22.3	0.21	2.47	29.9	0.20	15.2
**19**	8.96	6.91	41.3	0.36	6.87	20.8	0.31	12.8
**20**	13.6	6.12	33.0	0.35	6.52	17.1	0.42	13.9
**21**	10.6	19.7	24.6	0.44	9.86	5.74	0.43	16.6
**22**	12.2	12.4	26.4	0.46	9.00	3.08	0.30	18.0
**23**	11.3	8.68	30.1	0.31	5.77	15.7	0.29	14.9
**24**	10.4	11.8	23.7	0.30	6.49	13.2	0.34	15.8
**25**	18.3	12.2	56.2	0.70	12.7	10.2	0.48	13.9
**26**	10.9	3.91	17.0	0.33	4.29	16.2	0.22	12.1
**27**	11.0	3.10	11.5	0.21	8.46	31.2	0.26	13.0
**28**	11.0	3.51	13.0	0.32	7.35	30.6	0.30	14.5
**29**	11.3	9.20	18.6	0.39	6.21	10.3	0.12	15.9
**30**	11.6	2.98	13.3	0.34	5.62	30.9	0.42	14.6
**31**	9.36	6.87	21.7	0.33	5.02	18.1	0.28	13.3
**32**	7.10	6.48	16.8	0.31	4.72	26.0	0.49	14.0
**33**	10.5	4.81	28.9	0.33	4.41	27.1	0.63	14.1
**34**	7.70	13.8	21.6	0.30	6.73	14.2	0.47	14.3
**35**	4.90	26.4	15.4	0.33	5.57	17.8	0.38	14.2
**36**	9.46	6.37	20.9	0.36	6.14	32.9	0.28	14.7
**37**	9.30	21.5	26.3	0.38	6.71	7.39	0.26	15.1

*All concentration is in mg/kg dry weights, except for Fe which is in %.

The wet season starts from November to March while the dry season starts from May to September [4]. These seasonal patterns in Malaysia do influence the spatial distribution of heavy metals in the sediment of the Merang River. Since the wet season brings heavy rainfalls, the flood may occurs along the rivers especially at the upstream region. This flood may erode the riverbank [5] and increase the river flow and river discharge to the ocean. Thus a vast amount of pollutants such as heavy metals will also discharge during this season [6,7].

In this study, we found that the concentration of Li was higher in September 2017 (dry season). One possible reason that might affect the higher concentration especially in the river system was the runoff from the residential area. This runoff brings the contaminated soils into the river and ends up at the bottom of the river as sediment [8]. During the dry season, most of the chalets along the river and jetties were fully occupied.

## Experimental design, materials, and methods

Sampling was carried out in September and November 2017, January, March, May, and July 2018, subsequently every two months sampling period. A total of 64 stations were sampled, where 20 stations in the coastal area and 44 stations along the river area. Each station distance varies from 500 m to 1000 m, and the total distance of all sampling stations from the river up to coastal is approximately 8 km, where 2 km of the distance covers the coastal area of Merang River. The sampling area covers an area from 5.496900° to 5.50000°N latitude and 102.901700° to 102.942100°E longitude. Ponar Grab was used in the process of collecting surficial sediment in the study area. The sediment taken must not interact with the Ponar grab to avoid contamination were collected and put in polyethylene bag by using a plastic scope. The sediment samples collected were kept at low temperatures to minimize the sample degradation and transported back to the laboratory.

In the laboratory, the sediments were dried in an oven at 60 °C until a constant weight was reached. Then the sediments were ground with a porcelain pestle and mortar into a fine powder. In order to prevent contamination during the sample preparation and analysis, all glasswares were overnight immersed in the diluted 5% nitric acid (HNO_3_). After an overnight immersion, all the glassware were washed thoroughly using distilled water and dried in an oven [9].

A total of 50 mg of <63 µm mesh size sample was taken from each sample collected to be digested in a closed Teflon vessel with concentrated mixed acid of HCl, HNO_3,_ and HF with a ratio of 3:3:1 respectively. The Teflon Bomb digester was kept in an oven for 8 h at 100 °C. After that, the samples were left to cool down to room temperature. Then, the solution in each digester was thoroughly transferred in a 15 mL centrifuge tube and was diluted using Mili-Q water up to 10 mL. A clear solution with empty residue was seen and obtained at this stage. The concentrations of heavy metal elements were measured by using the Inductively Coupled Plasma Mass Spectrometry (ICP-MS) [10,11].

## Declaration of Competing Interest

The authors declare that there is no conflict of interest regarding the publication of this article. The authors also declare that they have no known competing for financial interests or personal relationships which have, or could be perceived to have, influenced the work reported in this article.

## Appendix Supplementary materials

Image, application 1
